# Supplementary material for: A Pragmatic Machine Learning Approach to Quantify Tumor-Infiltrating Lymphocytes in Whole Slide Images
Source: Cancers (Basel). 2022 Jun 16;14(12):2974. doi: 10.3390/cancers14122974 (PMC9221016; doi:10.3390/cancers14122974)
Supplement: Supplementary file 1 [file cancers-14-02974-s001.zip › cancers-1750993-supplementary.pdf]

Supplementary tables

Table S1. Clinicopathologic variables and their correlations with median dichotomized TILs identified using the PanNuke aug model (*n* = 87, chi-square and Fisher's exact tests).

			PanNuke Score		
	**N**	**(%)**	**Low**	**High**	**P**
Age					0.72
[0,65]	33	38%	18	15	
(65,85]	54	62%	26	28	
Gender					0.57
Female	21	24%	9	12	
Male	66	76%	35	31	
Smoking status					0.76
Never smoked	7	8%	4	3	
Present smoker	45	52%	24	21	
Previous smoker	35	40%	16	19	
ECOG					0.48
0	45	52%	20	25	
1	34	39%	20	14	
2	8	9%	4	4	
Histology					0.10
LUAD	35	40%	22	13	
LUSC	52	60%	22	30	
tStage					0.43
1	24	28%	15	9	
2	37	43%	18	19	
3	19	22%	9	10	
4	7	8%	2	5	
nStage					0.28
N0	55	63%	30	25	
N1	22	25%	8	14	
N2	10	11%	6	4	
pStage					0.06
I	33	38%	22	11	
II	35	40%	14	21	
III	19	22%	8	11	
Differentiation					0.47
Well	10	11%	7	3	
Moderate	34	39%	16	18	
Poor	43	49%	21	22	

Abbreviations: ECOG, Eastern Collaborative Oncology Group; LUAD, lung adenocarcinoma; LUSC, lung squamous cell carcinoma.

Table S2. HoVer-Net trained on the CoNSeP dataset (A I) and our reproduction (A II), and HoVer-Net trained on the PanNuke dataset (B I) and our replication (B II).

	**A)**		**B)**	
	**I**	**II**	**I**	**II**
	**CoNSeP original**	**CoNSeP reproduction**	**PanNuke**	**PanNuke reproduction**
Reference	(Graham *et al.*, 2019)		(Gamper *et al.*, 2020)	
Segmentation				
Dice2	0.85	0.84	NG	0.83
AJI	0.57	0.53	NG	0.67
DQ	0.70	0.66	NG	0.78
SQ	0.78	0.77	NG	0.61
PQ	0.55	0.50	NG	0.78
AJI+	NG	0.55	NG	0.68
Integrated Classification				
$F1_{overall}$	0.75	0.75	NG	0.80
$Accuracy_{overall}$	NG	0.83	NG	0.76
$Precision_{overall}$	NG	0.74	NG	0.79
$Recall_{overall}$	NG	0.75	NG	0.80
$F1_{inflammatory}$	0.63	0.52	0.54	0.50
$Precision_{inflammatory}$	NG	0.73	0.56	0.48
$Recall_{inflammatory}$	NG	0.40	0.51	0.53
$F1_{cancer}$	0.64	0.66	0.62	0.58
$Precision_{cancer}$	NG	0.59	0.58	0.57
$Recall_{cancer}$	NG	0.74	0.67	0.60

Table S3. A summary of the performance of four deep learning models trained using the CoNSeP (A I-II and B I-II) and the PanNuke (A III-IV and B III-IV) datasets using the original training pipeline as published by Graham et al. (Graham *et al.*, 2019) without (A I and III and B I and III) and with (A II and IV and B II and IV) enhanced augmentation (Graham *et al.*, 2019). The best results for each parameter 1) within each dataset are in bold and 2) for models trained on another dataset are in italics. Integrated classification results are in Table 2.

**Test data**	**CoNSeP**						**PanNuke**						**MoNuSAC**					
**Model**	**CoNSeP**		**PanNuke**		**MoNuAC**		**CoNSeP**		**PanNuke**		**MoNuSAC**		**CoNSeP**		**PanNuke**		MoNuAC	
Augmentation	HoVer	Aug	HoVer	Aug	HoVer	Aug	HoVer	Aug	HoVer	Aug	HoVer	Aug	HoVer	Aug	HoVer	Aug	HoVer	Aug
Numbering	AI	AII	AIII	AIV	AV	AVI	BI	BII	BIII	BIV	BV	BVI	CI	CII	CIII	CIV	CV	CVI
			Segmentation															
Dice2	**0.84**	**0.84**	*0.83*	0.82	0.56	0.55	0.62	*0.70*	**0.83**	**0.83**	0.60	0.62	0.50	0.61	0.70	*0.71*	0.74	**0.75**
AJI	**0.53**	**0.53**	*0.52*	0.51	0.32	0.32	0.42	*0.47*	0.67	**0.68**	0.45	0.46	0.33	0.41	*0.52*	*0.52*	0.56	**0.57**
DQ	**0.66**	0.65	0.63	*0.64*	0.41	0.41	0.50	0.55	**0.78**	**0.78**	0.57	*0.58*	0.42	0.50	0.68	*0.69*	0.75	**0.76**
SQ	**0.77**	0.50	0.48	*0.49*	0.32	0.32	0.32	0.38	0.61	**0.62**	0.41	0.42	0.30	0.38	0.52	*0.53*	0.60	**0.61**
PQ	0.50	0.77	0.76	0.77	***0.78***	***0.78***	0.63	0.68	0.78	**0.79**	*0.70*	*0.70*	0.61	0.70	0.75	*0.76*	0.79	**0.80**
AJI+	0.55	0.55	0.55	***0.56***	0.35	0.35	0.42	*0.48*	0.68	**0.69**	0.45	0.47	0.33	0.41	0.52	*0.53*	0.57	**0.58**
$Recall_d^{inflammatory}$	0.82	0.84	***0.89***	0.88	0.70	0.71	***0.91***	0.73	0.85	0.84	0.74	0.78	0.84	0.90	0.91	***0.93***	0.92	0.92
$Recall_d^{cancer}$	**0.78**	**0.78**	*0.74*	0.72	0.54	0.52	0.45	0.58	0.82	**0.83**	*0.62*	*0.62*	0.24	0.81	***0.97***	***0.97***	0.87	0.89
			Classification															
$Accuracy_c^{inflammatory}$	0.61	0.67	0.61	0.65	0.71	***0.72***	0.29	0.35	0.57	**0.59**	*0.48*	0.47	0.27	0.49	*0.79*	0.75	**0.87**	**0.87**
$Precision_c^{inflammatory}$	**0.85**	0.83	0.64	0.73	*0.84*	0.81	*0.66*	0.59	0.70	**0.76**	0.56	0.51	***0.94***	0.91	0.92	0.92	0.92	0.91
$Recall_c^{inflammatory}$	0.69	0.78	***0.93***	0.86	0.83	0.87	0.35	0.46	0.76	0.72	0.78	***0.85***	0.28	0.52	*0.84*	0.80	0.94	**0.95**
$F1_c^{inflammatory}$	0.76	0.80	0.76	0.79	0.83	***0.84***	0.45	0.51	0.73	**0.74**	*0.65*	0.64	0.43	0.66	0.88	*0.86*	**0.93**	**0.93**
$Accuracy_c^{cancer}$	0.88	**0.89**	*0.81*	0.79	0.70	0.73	0.38	0.44	0.72	**0.73**	*0.69*	0.68	0.15	0.45	*0.75*	0.73	**0.85**	**0.85**
$Precision_c^{cancer}$	0.90	**0.93**	*0.86*	0.81	0.72	0.76	0.89	***0.92***	0.83	0.80	0.80	0.83	***0.94***	***0.94***	0.78	0.75	0.87	0.88
$Recall_c^{cancer}$	**0.97**	0.94	0.93	***0.97***	0.96	0.95	0.40	0.46	0.83	**0.89**	*0.83*	0.79	0.15	0.46	*0.96*	0.96	**0.98**	0.97
$F1_c^{cancer}$	**0.94**	**0.94**	*0.89*	0.88	0.83	0.84	0.55	0.61	0.83	**0.84**	*0.82*	0.80	0.27	0.62	*0.86*	0.85	**0.92**	**0.92**

Table S4. A comparison of disease-specific survival of NSCLC patients according to high and low levels of TILs identified in H&E WSIs using different approaches (*n* = 87, univariable analyses, log-rank test).

	N(%)	5 Year	Median	HR(95%CI)	P
TILs in H&E WSIs: manual score					0.173
0-5%	9(10)	44	27	1.000	
6-25%	47(54)	51	114	0.8(0.24-2.66)	
26-50%	22(25)	76	NA	0.39(0.11-1.33)	
>50%	5(6)	80	NA	0.25(0.05-1.28)	
Missing	4(5)				
CD8+ cells in DAB stained TMAs: QuPath cell count					**0.003**
Low	41(47)	44	51	1.000	
High	41(47)	79	NA	0.34(0.17-0.68)	
Missing	5(6)				
TILs in H&E WSIs: rule based approach					0.095
Low	44(51)	54	114	1.000	
High	43(49)	69	NA	0.56(0.29-1.1)	
TILs in HE WSIs: HoVer-Net CoNSeP Aug Model					**0.013**
Low	44(51)	51	64	1.000	
High	43(49)	70	NA	0.42(0.22-0.84)	
TILs in HE WSIs: HoVer-Net CoNSeP Orig Model					0.088
Low	44(51)	55	83	1.000	
High	43(49)	67	NA	0.55(0.28-1.08)	
TILs in HE WSIs: HoVer-Net MoNuSAC Aug Model					**<0.001**
Low	44(51)	44	47	1.000	
High	43(49)	77	NA	0.27(0.14-0.53)	
TILs in HE WSIs: HoVer-Net MoNuSAC Orig Model					**<0.001**
Low	44(51)	48	47	1.000	
High	43(49)	73	NA	0.35(0.18-0.69)	
TILs in HE WSIs: HoVer-Net PanNuke Aug Model					**<0.001**
Low	44(51)	44	47	1.000	
High	43(49)	77	NA	0.3(0.15-0.6)	
TILs in HE WSIs: HoVer-Net PanNuke Orig Model					**0.007**
Low	44(51)	49	51	1.000	
High	43(49)	72	NA	0.39(0.2-0.76)	

Abbreviations: TIL, tissue infiltrating lymphocyte; CD, cluster of differentiation;

Table S5. Manual estimation of precision and recall and calculated F1 scores for cancer- and immune cells on the output of the PanNuKe, CoNSeP and MoNuSAC models with updated augmentation. The 20 1000x1000µm patches were randomly sampled from our lung cancer cohort.

	**PanNuke aug**						**CoNSeP aug**						**MoNuSAC aug**					
	**Cancer cells**			**Immune cells**			**Cancer cells**			**Immune cells**			**Cancer cells**			**Immune cells**		
Slide	Rec	Prec	F1	Rec	Pre	F1	Rec	Prec	F1	Rec	Pre	F1	Rec	Prec	F1	Rec	Pre	F1
1	0.97	0.80	0.88	0.80	0.97	0.88	0.97	0.80	0.88	0.90	0.97	0.93	0.97	0.90	0.93	0.80	0.97	0.88
2	0.97	0.80	0.88	0.80	0.97	0.88	0.97	0.80	0.88	0.90	0.97	0.93	0.98	0.75	0.85	0.80	0.97	0.88
3	0.97	0.85	0.91	0.70	0.97	0.81	0.97	0.80	0.88	0.80	0.97	0.88	0.97	0.90	0.93	0.80	0.97	0.88
4	0.97	0.80	0.88	0.70	0.97	0.81	0.97	0.80	0.88	0.80	0.97	0.88	0.97	0.80	0.88	0.80	0.97	0.88
5	0.97	0.30	0.46	0.50	0.97	0.66	0.97	0.30	0.46	0.50	0.97	0.66	0.97	0.55	0.67	0.80	0.97	0.88
6	0.97	0.30	0.46	0.10	0.97	0.18	0.97	0.20	0.33	0.10	0.97	0.18	0.97	0.60	0.74	0.70	0.97	0.81
7	0.97	0.10	0.18	0.20	0.97	0.33	0.97	0.10	0.18	0.30	0.97	0.46	0.97	0.30	0.46	0.60	0.97	0.74
8				0.20	0.97	0.17				0.30	0.97	0.46				0.50	0.97	0.66
9	0.97	0.30	0.46	0.30	0.97	0.46	0.97	0.30	0.46	0.30	0.97	0.46	0.97	0.50	0.66	0.50	0.97	0.66
10	0.97	0.80	0.88	0.80	0.97	0.88	0.97	0.80	0.88	0.70	0.97	0.81	0.97	0.90	0.93	0.55	0.97	0.70
11	0.97	0.50	0.66	0.50	0.97	0.66	0.97	0.50	0.66	0.50	0.97	0.66	0.97	0.50	0.66	0.40	0.97	0.57
12	0.97	0.60	0.74	0.50	0.97	0.66	0.97	0.50	0.66	0.40	0.97	0.57	0.97	0.40	0.57	0.40	0.97	0.57
13	0.97	0.70	0.81	0.40	0.97	0.57	0.97	0.70	0.81	0.40	0.97	0.57	0.97	0.75	0.85	0.80	0.97	0.88
14	0.97	0.80	0.88	0.60	0.97	0.74	0.97	0.80	0.88	0.60	0.97	0.74	0.97	0.75	0.85	0.80	0.97	0.88
15	0.97	0.80	0.88	0.40	0.97	0.57	0.97	0.70	0.81	0.30	0.97	0.46	0.97	0.55	0.69	0.50	0.97	0.66
16	0.97	0.80	0.88	0.40	0.97	0.57	0.97	0.70	0.81	0.50	0.97	0.66	0.97	0.50	0.66	0.40	0.97	0.57
17	0.97	0.80	0.88	0.80	0.97	0.88	0.97	0.80	0.88	0.70	0.97	0.81	0.97	0.90	0.93	0.80	0.97	0.88
18	0.97	0.70	0.81	0.20	0.97	0.33	0.97	0.70	0.81	0.20	0.97	0.33	0.97	0.40	0.57	0.30	0.97	0.46
19	0.97	0.70	0.81	0.30	0.97	0.46	0.97	0.70	0.81	0.30	0.97	0.46	0.97	0.40	0.57	0.40	0.97	0.57
20	0.97	0.80	0.88	0.30	0.97	0.46	0.97	0.70	0.81	0.30	0.97	0.46	0.97	0.60	0.74	0.30	0.97	0.46
Med	0.97	*0.64*	***0.75***	*0.48*	0.97	*0.60*	0.97	0.62	0.72	*0.49*	0.97	*0.62*	0.97	*0.63*	*0.74*	***0.60***	0.97	***0.72***
Mean	0.97	*0.80*	***0.88***	*0.45*	0.97	*0.61*	0.97	*0.70*	*0.81*	*0.45*	0.97	*0.61*	0.97	0.60	0.74	***0.58***	0.97	***0.72***
Min	0.97	0.10	0.18	*0.10*	0.97	*0.17*	0.97	0.10	0.18	0.10	0.97	0.18	0.97	0.30	**0.46**	***0.30***	0.97	***0.46***
Max	0.97	*0.85*	*0.91*	0.80	0.97	0.88	0.97	0.80	0.88	**0.90**	0.97	**0.93**	0.98	0.90	**0.93**	*0.80*	0.97	*0.88*
Range	0.00	*0.75*	*0.72*	*0.70*	0.00	*0.71*	0.00	0.70	0.70	0.80	0.00	0.75	0.01	**0.60**	**0.48**	***0.50***	0.00	***0.42***

Abbreviations: Rec, recall; Prec, precision

Table S6. Manual estimation of precision and recall and calculated F1 scores for cancer- and immune cells on the output of the PanNuKe, CoNSeP and MoNuSAC models with original augmentation. The 20 1000x1000µm patches were randomly sampled from our lung cancer cohort.

	**PanNuke original**						**CoNSeP original**						**MoNuSAC original**					
	**Cancer cells**			**Immune cells**			**Cancer cells**			**Immune cells**			**Cancer cells**			**Immune cells**		
Slide	Rec	Prec	F1	Rec	Pre	F1	Rec	Prec	F1	Rec	Pre	F1	Rec	Prec	F1	Rec	Pre	F1
1	0.96	0.80	0.87	0.76	0.97	0.85	0.97	0.85	0.91	0.70	0.97	0.81	0.97	0.85	0.91	0.70	0.97	0.81
2	0.96	0.80	0.87	0.80	0.97	0.88	0.97	0.79	0.87	0.60	0.97	0.72	0.98	0.75	0.85	0.60	0.97	0.74
3	0.96	0.78	0.85	0.70	0.97	0.81	0.97	0.85	0.91	0.70	0.89	0.78	0.97	0.90	0.93	0.70	0.97	0.81
4	0.96	0.80	0.87	0.75	0.97	0.85	0.97	0.80	0.88	0.75	0.97	0.85	0.97	0.70	0.81	0.65	0.97	0.78
5	0.97	0.29	0.44	0.40	0.97	0.57	0.97	0.60	0.72	0.45	0.97	0.53	0.97	0.55	0.67	0.70	0.97	0.81
6	0.97	0.20	0.33	0.10	0.97	0.18	0.96	0.40	0.54	0.40	0.97	0.50	0.97	0.60	0.74	0.65	0.97	0.78
7	0.97	0.10	0.18	0.08	0.97	0.14	0.97	0.20	0.32	0.40	0.97	0.54	0.97	0.30	0.46	0.50	0.97	0.66
8				0.20	0.97	0.17				0.50	0.97	0.66				0.40	0.97	0.57
9	0.97	0.20	0.33	0.30	0.97	0.46	0.97	0.40	0.56	0.35	0.97	0.50	0.97	0.50	0.66	0.45	0.97	0.61
10	0.97	0.80	0.88	0.80	0.97	0.88	0.97	0.85	0.91	0.60	0.97	0.74	0.97	0.80	0.88	0.50	0.97	0.65
11	0.97	0.45	0.61	0.50	0.97	0.66	0.97	0.45	0.61	0.35	0.97	0.51	0.97	0.50	0.66	0.30	0.97	0.46
12	0.97	0.50	0.65	0.50	0.97	0.66	0.97	0.40	0.57	0.25	0.97	0.37	0.97	0.40	0.57	0.30	0.97	0.46
13	0.96	0.70	0.81	0.40	0.97	0.57	0.97	0.70	0.81	0.58	0.97	0.70	0.97	0.70	0.81	0.65	0.97	0.78
14	0.96	0.80	0.87	0.50	0.97	0.66	0.97	0.75	0.85	0.45	0.97	0.53	0.97	0.75	0.85	0.73	0.97	0.83
15	0.97	0.80	0.88	0.40	0.97	0.57	0.97	0.55	0.69	0.38	0.97	0.53	0.97	0.55	0.69	0.40	0.97	0.57
16	0.97	0.80	0.88	0.35	0.97	0.51	0.97	0.65	0.77	0.30	0.97	0.45	0.97	0.50	0.66	0.35	0.97	0.51
17	0.97	0.80	0.88	0.80	0.97	0.88	0.97	0.85	0.91	0.75	0.97	0.85	0.97	0.80	0.88	0.70	0.97	0.81
18	0.97	0.70	0.81	0.20	0.97	0.33	0.97	0.55	0.69	0.23	0.97	0.36	0.97	0.40	0.57	0.20	0.97	0.33
19	0.97	0.70	0.81	0.30	0.97	0.46	0.97	0.55	0.69	0.25	0.97	0.37	0.97	0.40	0.57	0.35	0.97	0.51
20	0.97	0.70	0.81	0.33	0.97	0.49	0.97	0.70	0.81	0.25	0.97	0.39	0.97	0.60	0.74	0.20	0.97	0.33
Med	0.97	0.62	0.72	0.46	0.97	0.58	0.97	0.63	0.74	0.46	0.97	0.58	0.97	0.61	0.73	0.50	0.97	0.64
Mean	0.97	0.70	0.81	0.40	0.97	0.57	0.97	0.65	0.77	0.43	0.97	0.53	0.97	0.60	0.74	0.50	0.97	0.66
Min	0.96	0.10	0.18	0.08	0.97	0.14	0.96	0.20	0.32	0.23	0.89	0.36	0.97	0.30	0.46	0.20	0.97	0.33
Max	0.97	0.80	0.88	0.80	0.97	0.88	0.97	0.85	0.91	0.75	0.97	0.85	0.98	0.90	0.93	0.73	0.97	0.83
Range	0.02	0.70	0.70	0.73	0.00	0.74	0.01	0.65	0.59	0.53	0.09	0.49	0.01	0.60	0.48	0.53	0.00	0.50

Abbreviations: Rec, recall; Prec, precision

Supplementary figures

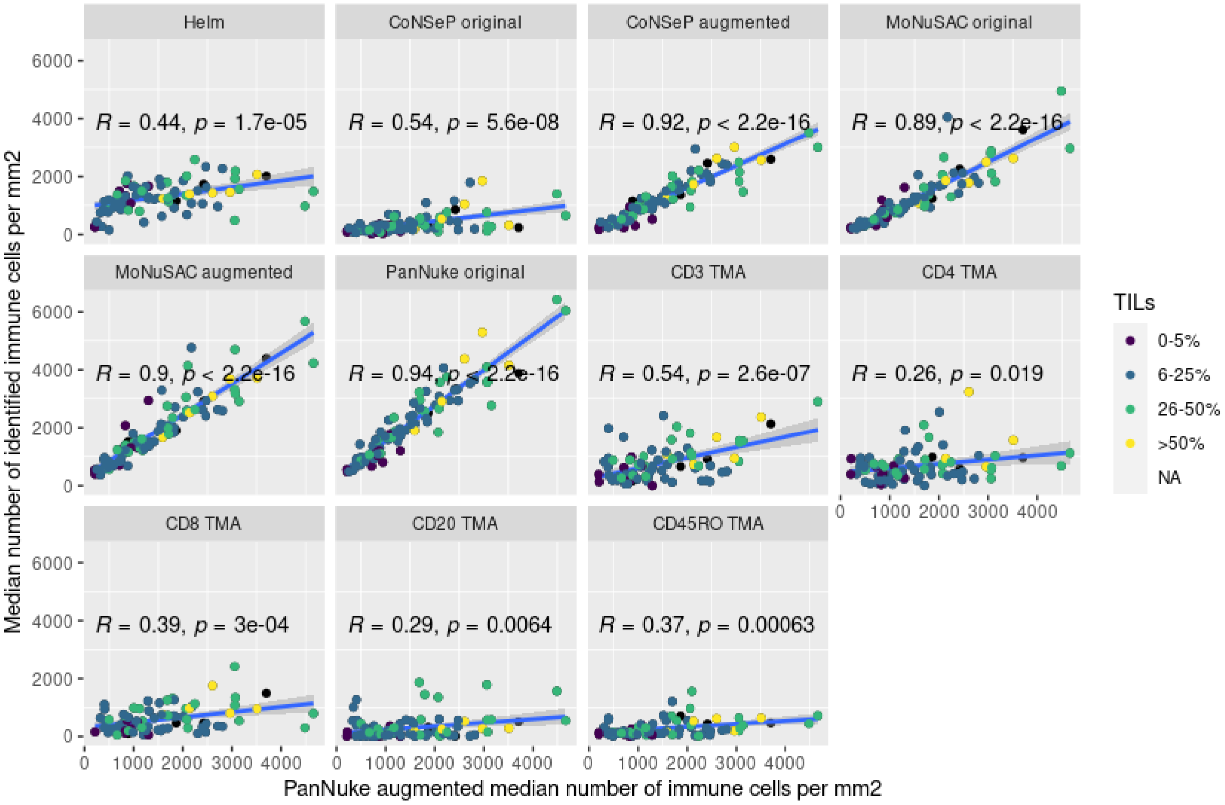

Figure S1. Median number of immune cells for each patient identified using the augmented PanNuke model plotted against the median number of immune cells identified by: 1) HeIm, CoNSeP original (HoVer-Net paper), CoNSeP augmented, MoNuSAC original, MoNuSAC augmented and PanNuke original, using the dataset provided in this publication and 2) CD3, CD4, CD8, CD20 and CD45RO identified on TMAs for each patient (the full TMA results are published (Kilvaer *et al.*, 2020). Each dot represents data from an individual patient and is labelled according to the semi-quantitative TILs score he/she was assigned in (Rakaee *et al.*, 2018).

Supplementary materials and methods

HeIm: A simple rule based algorithm

We implement a simple rule based approach for annotating cells as a baseline for automated approaches. We first do simple image augmentation by converting from the RGB to the HED color space using color deconvolution as described above. Then we generate a mask thresholding each channel in the HED color space. The mask is then morphologically opened using an elliptical and then a square kernel. This results in two masks.

We apply *contour detection* and filtering to ensure that only detections within the tolerated size and circularity range are kept. We remove duplicates using overlap detection. Finally, we set the ranges of the hematoxylin and eosin image channel for the mask to 220-255 and 0-50, the range of the area to 190-600px^2 and the minimum circularity to 65. The source code is in: https://github.com/uit-hdl/HEImmune.

Definition of metrics used in the replication study

To compare our results with those previously published by Graham et al, (Graham *et al.*, 2019) we used the custom defined F-score for different types of cells they propose in their paper.

$F_{dcr}^{t} = \frac{2(TP_{dcr}^{t}+TN_{dcr}^{t})}{2(TP_{dcr}^{t}+TN_{dcr}^{t})+\alpha_0 FP_{dcr}^{t}+\alpha_1 FN_{dcr}^{t}+\alpha_2 FP_d+\alpha_3 FN_d}$ where TP_{dcr}^{t} is correct instances classifications of type t, TN_{dcr}^{t} as correctly classified instances of types other than type t, FN_{dcr}^{t} as incorrectly classified instances of types other than type t, FP_{dcr}^{t} as incorrectly classified instances of type t, FN_d as misdetected GT instances and FP_d as overdetected predicted instances, $\alpha_0 = \alpha_1 = 2$ and $\alpha_2 = \alpha_3 = 1$ for taking into account detection results, with higher emphasis on classification performance.

In order to get a deeper understanding of the models performance we introduced precision ($Precision_{dcr}^{t}$) and recall ($Recall_{dcr}^{t}$) scores, based on the custom defined F-score defined above, that encompass detection (X_d) and classification (X_c) results. We use $TPN = TP_{dcr}^{t} + TN_{dcr}^{t}$, $FP = 2 \cdot FP_{dcr}^{t} + FP_d$, $FN = 2 \cdot FN_{dcr}^{t} + FN_d$, to calculate $Precision_{dcr}^{t} = \frac{TPN}{TPN+FP}$ and $Recall_{dcr}^{t} = \frac{TPN}{TPN+FN}$. This latter approach is similar to Gamper et al. (Gamper *et al.*, 2020) and allows comparisons with models they built using the HoVer-Net infrastructure and PanNuke data.

Definition of metrics used to separately evaluate segmentation and classification

Segmentation metrics include:

$DICE = \frac{2TP}{2TP+FP+FN}$, $DICE2$computes and aggregates $DICE$per nucleus.

$AJI = \frac{\sum_{i=1}^{N}|G_i \cap P_i|}{\sum_{i=1}^{N}|G_i \cap P_i| + \sum_{i \in rest}|P_i|}$, where $P_i$is the predicted nucleus that maximizes the Jaccard Index with the ground truth nucleus G_i and $rest$ refers to the collection of P_i with no match.

$$DQ = \frac{|TP|}{|TP| + 0.5|FP| + 0.5|FN|}$$

$SQ = \frac{\sum_{(x,y)\in TP} IOU(x,y)}{|TP|}$, $IOU(x,y)$ - intersection over union between x and y sets

$$PQ = DQ \times SQ$$

$Recall_d^t = \frac{TP_d^t}{TP_d^t + FN_d^t}$, where TP_d^t - detected cells with GT label t, FN_d^t- cell with GT label t not detected.

Classification metrics include:

$Accuracy_c^t = \frac{TP_c^t + TN_c^t}{TP_c^t + TN_c^t + FP_c^t + FN_c^t}$, $Precision_c^t = \frac{TP_c^t}{TP_c^t + FP_c^t}$, $Recall_c^t = \frac{TP_c^t}{TP_c^t + FN_c^t}$, $F1_c^t = \frac{TP_c^t}{TP_c^t + 0.5(FP_c^t + FN_c^t)}$, where

TP_c^t- detected cells with GT label t, classified as t,

TN_c^t- detected cells with GT label other than t, classified as other than t,

FP_c^t- detected cells with GT label other than t, classified as t,

FN_c^t- detected cells with GT label t classified as other than t, for each class t.

Supplementary results

Model training and inference times

Training times for the CoNSeP, PanNuke, and MoNuSAC datasets were 30h, 60h and 31h on the Azure cloud and 11h, 38h and 10h on the local workstation, respectively. The cloud enables horizontal scaling for the inference part, thus time for the post processing step may be reduced, depending on available resources. Inference times on the UiT-TILs dataset were 27h and 12h corresponding to 81s and 36s for single patches, for Azure and workstation, respectively. Based on the size of the patches, we estimate that inference and post processing time for typical WSIs (10x15mm) is between 1h30min and 3h30min on our current Azure and workstation deployments.
